# A Few Notes on Foreign Bodies Swallowed by Cattle

**Published:** 1899-07

**Authors:** Arthur S. Wheeler

**Affiliations:** Biltmore Farms, Biltmore, N. C.


### A FEW NOTES ON FOREIGN BODIES SWALLOWED BY CATTLE.

By Arthur S. Wheeler, V.M.D.,
BILTMORE FARMS, BILTMORE, N. C.

From time to time there appear in our veterinary literature reports of cases of traumatic pericarditis in the cow, most commonly due to swallowing nails or fragments of baling-wire, which pass from the digestive tract (reticulum) through the diaphragm into the pericardial sac.

In the indispensable Friedberger and Frohner's *Pathology and Therapeutics* a short chapter is devoted to "Gastric Derangement from Foreign Bodies, or Traumatic Indigestion;" and a bulletin on traumatic inflammation of the stomach and diaphragm has been published by the United States Department of Agriculture under the title of "Injuries to Cattle from Swallowing Pointed Objects," by Drs. Smith and Dawson. But the meagreness of investigations on the rôle played by foreign bodies in inducing disease, especially in dairy cattle, leads me to present a few memoranda of post-mortems bearing upon this topic.

From the superficial observations which I have made in slaughter-houses, where the histories of the cases are the unknown quantity, and from my experience in treating the diseases of dairy cattle, I am inclined to the belief that foreign bodies are responsible for a great many more evils than pericarditis and indigestion. Whoever has examined the lesions of liver, spleen, peritoneum, lungs, heart, uterus, in the form of every possible variety of adhesions of one organ to another, the formation of neoplastic tissue and modifications of structure, the multiple abscesses almost rivalling those of tuberculosis, cannot fail to be struck by the importance of the subject. That these foreign bodies, which are generally blamed for traumatic pericarditis alone, may excite almost any train of symptoms seems to me highly probable, as reflex nyphomania, secondary metritis, septicæmia, etc. If I succeed in invoking the closer attention of the profession to what seems to me a very significant factor in the health and welfare of our dairy herds, the mission of the following brief notes will be fulfilled.

The first four cases died, or were destroyed, the remainder were butchered for various causes.

Case I.—Cow: After calving did not shed secundines, which were removed artificially. Had a foul discharge from vulva soon afterward; also intermittent fever, appetite irregular, in fact, not well after calving. Went down steadily in her milk, discharge from womb became purulent; cow became emaciated and cachectic, and was finally destroyed as an act of humanity.

Necropsy revealed a pint of creamy pus in the uterus; os dilated; whole generative apparatus inflamed; both ovaries cystic; numerous abscesses from size of pea to walnut along serous surface of uterus. On serous coats of large and small intestines were festoons of abscesses; intestines, paunch, and uterus attached to one another by fibrous and inflammatory products; mediastinal glands enlarged and containing pus. Liver contained a few nodules deep in the organ, and a few pearly spots on surface. There was a gelatinous tumor on reticulum, and, although no foreign body was found in it, the blackened channel of a piece of metal could be distinctly traced out. The pus of all the abscesses was characterized by having a greenish tint and a very offensive odor.

This cow failed to react to the tuberculin test.

Case II.—This cow was ailing for two weeks; no predominant symptoms. There was a gradual wasting away. Everything seems to be wrong with her, and yet there was nothing specifically wrong; intermittent fever, capricious appetite, irregular bowels; in short, a veritable cachexia, and very much the same as Case I., except the vaginal discharge; nor do my notes show that this cow had recently calved.

Necropsy showed many nails and stones in the reticulum, one of the nails penetrating the wall of the same, forming on the outer surface the characteristic hard, fibrous, gelatinous tumor, which was the focus of all manner of adhesions among the viscera. There were countless abscesses, especially on the liver and spleen, and all filled with foul-smelling pus and débris of the consistency of cement. The liver weighed twenty-four pounds, its hepatic vein containing this same plastic pus; it was proliferated and cirrhosed, the hepatic ducts being indurated and thickened, their lumen almost obliterated. The changes in the liver were much the same as those of the flukey liver, of which I used to throw away about fifteen hundred dollars' worth a year in the Crescent City slaughter-house. There was an abscess behind the left shoulder-blade in the muscular tissue, containing a cupful of foul-smelling pus.

This cow was tested for tuberculosis, but gave no reaction, nor did the necropsy reveal the characteristic changes of this disease.

Case III.—This cow was sick for two months, but I did not see her until about two weeks before she died. She died by inches—was tested for tuberculosis four days before she died, giving her ten c.c., or three times the usual dose of tuberculin, and obtaining no reaction.

Necropsy showed two finishing-nails embedded in the floor of the abdomino-thoracic cavity, and in the latter cavity there was an enormous abscess containing about two gallons of fetid pus and débris, and in addition to this there was about three gallons of serum in the thoracic cavity; there was also an œdematous swelling from sternum to maxillary region along lower border of neck, which appeared a few days before death, and contained serum. There was considerable adhesion between the lungs and pericardial membrane, more or less consolidation of lungs and pericarditis, which accounted for distress of breathing.

Case IV.—This cow took the bull August 17, 1897, being very much excited at the time. She was slightly off her feed the same evening. Gave her dose of salts which had no effect until evening, August 18th, and not then until she had received an enema. Her temperature was 103.5°, 10 A.M., 18th; pulse 80; lack-lustre expression in eyes, and staggering gait.

Morning 19th, much worse in every way. By placing hand in rectum liquid manure would run out, seemed to have nearly lost power of controlling sphincter-ani. She was down all day the 19th, dull, and no response to pin-pricks, becoming comatose, and finally convulsions alternating with coma; could swallow liquids from a bottle, but in a mechanical way; temperature 101°–102° on the 19th, and died about midnight of 19th.

*Necropsy:* Left-hand quarter of udder obliterated, containing a quantity of serum in its sinuses; this quarter had been obsolete for several years. There was a ten-penny wire nail projecting through reticulum about an inch, and the tumor induced by same was attached to floor of abdomen; the track of the nail formed a channel in the tumor that was stained black. There was an abscess the size of a fist on lower surface of paunch, and many fibrinous attachments of paunch and walls of abdomen; also considerable pleuritic effusion and chronic pleuritic changes. There was another abscess on right horn of uterus about the size of a walnut, and several decolorized patches on surface of liver. The pericardium showed ecchymotic spots, considerable fluid in sac, and myocardium showed inflammation. Uterus contained brownish friable clots of débris, and was somewhat congested (probably from recent rutting and service).

This cow had a severe attack of indigestion in April preceding fatal illness. There was obstinate constipation and impaction of ten days' duration, during which time she did not have one good action of the bowels and did not partake of one good feed. She calved July 10th, the calf dying in a few hours, being puny and scouring from first.

The following were butchered for various causes:

Case V.—Heifer: Cysts in both ovaries; two small yellowish patches on surface of liver, and an abscess in interior containing greenish pus. Slight peritoneal adhesions. The usual lump, size of two fists, containing three-inch piece of wire. Lungs slightly consolidated in neighborhood of diaphragm, and marbled.

Case VI.—Cow: One ovary converted into a cyst in its entirety, and as big as an English walnut, the other ovary likewise contained cysts. Some pleurisy present, due to nail-puncture of reticulum, and in addition there were four other ten-penny nails threaded through folds of reticulum. Large fibro-gelatinous tumor. Liver adherent by false bands to diaphragm. Considerable metritis.

Case VII.—Cow: Persistent buller for over a year and unable to breed. Inflammation of vagina, some metritis. Both ovaries practically converted into cysts, one cyst being as large as a hen's egg.

One eight-penny finishing-nail, one eight-penny cut-nail, two fragments of No. 12 iron wire (one piece three inches long, the other four inches), all through the wall of the reticulum, causing a large fibrous lump, size of a cocoanut, and the attachment of reticulum to diaphragm. Omentum adherent to side of abdominal wall.

Case. VIII.—Perpetual buller for year or more. The worst case I ever saw. She had large hygromas on knees from dropping on them prior to mounting an imaginary cow. The expression of her eyes was wild, her neck became fleshy, from constantly using the muscles of the neck, and even her voice became bull-like. Both ovaries were cystic, one being almost obliterated by cysts, one ovary contained a small Graafian vesicle. There was some metritis and leucorrhœa. Two-inch piece of wire formed a diverticulum in wall of reticulum. Also eight-penny finishing-nail and a brass pin in folds of same. There was the usual hard tumor overlying the above-mentioned cul-de-sac. Also attachments of tumor to diaphragm and liver to diaphragm. Two abscesses size of bird's egg on liver.

Case IX.—Cow: Perpetual buller; slight inflammation of vagina and metritis. Ovaries contain cysts, also Graafian vesicle in one ovary. Some adhesions in abdominal cavity, and a waxy lump in mesentery. Liver had false attachment, small lump over reticulum, but could find no foreign body.

Case X.—Cow: One quarter of udder indurated and atrophied. Five months' fœtus; lump size of two fists in thoracic cavity corresponding to point of contact of reticulum and diaphragm; this lump showed the stained tracks of foreign bodies in its interior. There were two pieces of wire two and one-half inches long, a ten-penny nail, and a four-inch piece of flat iron, all more or less through the wall of reticulum—spleen showed red inflamed spots throughout its structure, but especially along border.

Case XI.—Chronic buller; slight metritis in one cornu, and multiple cysts in corresponding ovary; the other ovary contained two Graafian vesicles. Some adhesions in abdominal cavity.

There were seventeen foreign bodies in the form of fragments of wire and nails, one staple and a piece of stone in the reticulum, but none of these had punctured the walls, being mostly short and blunt. The search for these bodies in this case being only incidental, my notes do not contain anything further.

Case XII.—Cow: Reacted to tuberculin test and showed unmistakable lesions of tuberculosis.

She contained nine pieces of metal in reticulum—one ten-penny wire-nail was through the walls of reticulum, producing on its outer surface the characteristic gelatinous tumor, which latter also contained some pus (non-tuberculous).

Case XIII.—Young eighteen months' bull: Pin through wall of reticulum and producing a purulent channel.

Case XIV.—Chronic buller for eight months; both ovaries cystic; two large gelatinous tumors on reticulum. There were also the following inside of same, lying loose: four nails, one being two and one-half inches long; seven pieces of wire, the longest being two and one-half inches; fifteen bits of metal, one being a link of endless chain.

Case XV.—Old steer: (Having to remove many of the organs from the offal-box, and some of them being hacked by the butcher, my notes are not as complete on this case as I should wish).

Liver almost unrecognizable as to shape, color, and structure, and containing débris and foul-smelling creamy pus and hard fibrous lumps, all due, in my opinion, to foreign bodies. The reticulum, which was mutilated, showed several cul-de-sacs in its walls, and innumerable pieces of wire.

Case XVI.—Cow: Suffered over a year with urinary trouble, and, prior to her destruction, for a month had frequent attacks of hæmaturia.

Kidneys, uterus, and bladder showed most profound inflammation and induration, the walls of whole urinary apparatus much thickened and filled with débris and blood-clots. Seven fragments of metal, wire-nails, etc., found in reticulum, and a three-inch piece of wire through the folds of same.

Case XVII.—Cow: Abscess of liver, at which point it was firmly attached to diaphragm; the abscess contained fetid pus of a greenish tint, and caused by a three-inch piece of baling-wire from the reticulum. There were also seven or eight scars in reticulum, showing where other injuries had occurred to that organ by foreign bodies.

Case XVIII.—Cow: Twenty years of age; right lung, lower portion, adherent to wall of thoracic cavity from sixth rib forward, and left lung from third rib forward. Three-and-a-half inch piece of wire (bent 135°) embedded in gelatino-connective tissue, tumor in the notch between the anterior and posterior lobules to the right of median line. There was more or less thickening of the lung-tissue itself in that region, and consolidation. A ten-penny wire-nail in a cul-de-sac of reticulum—innumerable cicatrices in reticulum, and one particularly large empty cul-de-sac that must have been the temporary resting-place of the wire, before it migrated to the thoracic cavity. The septa of the reticulum full of holes. [*Note.*—This cow had swelling over its body, right side, about a year before, which gradually disappeared. At the time I was unable to account for it. The migration of this wire may explain it. The piece of wire was considerably corroded and had a gritty deposit on its surface; it may have been in the cow for years.]

SUMMARY.—Of forty-two animals examined for foreign bodies, thirty-two had them to a greater or less degree. Out of eighteen of the most pronounced cases, four died directly or indirectly from their presence. In sixteen the foreign bodies had punctured the walls of the reticulum. Unless these bodies are sufficiently pointed, or long enough (two or more inches) they seldom puncture the wall of the reticulum. Wire-nails, especially finishing-nails and baling-wire, are the most dangerous. It is astonishing to what a degree these foreign-body lesions may be developed before there is any outward manifestation, and then the outward signs do not seem to be commensurate with the changes that have taken place. There is a strong probability that these bodies may migrate back into the reticulum, or become broken or modified by chemical action, thereby leaving only cicatricial tissue to mark their presence.

